# Familial perimesencephalic subarachnoid hemorrhage: two case reports

**DOI:** 10.1186/1752-1947-8-380

**Published:** 2014-11-22

**Authors:** Ulaş Cıkla, Beverly Aagaard-Kienitz, Patrick A Turski, Guner Menekse, David B Niemann, Mustafa K Başkaya

**Affiliations:** 1 Department of Neurological Surgery, School of Medicine and Public Health, University of Wisconsin, Madison, WI, USA; 2 Department of Radiology, University of Wisconsin School of Medicine and Public Health, Madison, WI, USA; 3 Department of Neurological Surgery, University of Wisconsin-Madison, School of Medicine, CSC, K4/822, 600 Highland Avenue, Madison, WI 53792, USA

**Keywords:** Cerebral angiography, Familial subarachnoid hemorrhage, Non-aneurysmal subarachnoid hemorrhage, Perimesencephalic subarachnoid hemorrhage

## Abstract

**Introduction:**

Non-aneurysmal spontaneous subarachnoid hemorrhage is characterized by an accumulation of a limited amount of subarachnoid hemorrhage, predominantly around the midbrain, and a lack of blood in the brain parenchyma or ventricular system. It represents 5% of all spontaneous subarachnoid hemorrhage cases. In spite of extensive investigation, understanding of the mechanisms leading to perimesencephalic non-aneurysmal subarachnoid hemorrhage remains incompletely defined. A growing body of evidence has supported a familial predisposition for non-aneurysmal spontaneous subarachnoid hemorrhage.

**Case presentation:**

A 39-year-old Caucasian man presented with sudden onset headache associated with diplopia. His computed tomography scan revealed perimesencephalic subarachnoid hemorrhage. A cerebral angiogram showed no apparent source of bleeding. He was treated conservatively and discharged after 1 week without any neurological deficits. The older brother of the first case, a 44-year-old Caucasian man, presented 1.5 years later with acute onset of headache and his computed tomography scan also showed perimesencephalic non-aneurysmal subarachnoid hemorrhage. He was discharged home with normal neurological examination 1 week later. Follow-up angiograms did not reveal any source of bleeding in either patient.

**Conclusions:**

We report the cases of two siblings with perimesencephalic non-aneurysmal subarachnoid hemorrhage, which may further suggest a familial predisposition of non-aneurysmal spontaneous subarachnoid hemorrhage and may also point out the possible higher risk of perimesencephalic non-aneurysmal subarachnoid hemorrhage in the first-degree relatives of patients with perimesencephalic non-aneurysmal subarachnoid hemorrhage.

## Introduction

Spontaneous subarachnoid hemorrhage (SAH) is a devastating clinical problem and is most commonly caused by a rupture of an intracranial aneurysm. In approximately 15% of patients with spontaneous SAH, the origin of the hemorrhage cannot be detected, despite extensive diagnostic imaging studies, and this is called non-aneurysmal subarachnoid hemorrhage (NSAH) [1,2]. NSAH can be subdivided into two different subgroups: perimesencephalic non-aneurysmal subarachnoid hemorrhage (P-NSAH) and non-perimesencephalic non-aneurysmal subarachnoid hemorrhage (nP-NSAH). P-NSAH is characterized by accumulation of subarachnoid blood, predominantly around the midbrain, and a lack of blood in the brain parenchyma or ventricular system [1,2]. In contrast, computed tomography (CT) scans of patients with nP-NSAH reveal bleeding patterns similar to those seen in aneurysmal SAH.

P-NSAH has generally been considered a distinct entity after the identification of different prognoses from the other NSAH by van Gijn *et al.*[2]. Patients with P-NSAH not only have a much lower risk of morbidity and mortality than the patients with aneurysmal SAH, but also have better outcomes than patients with nP-NSAH [1-3].

It has repeatedly been shown that genetic factors play an important role in the pathogenesis of SAH [4,5]. An apparent relationship between genetic factors and P-NSAH has not been defined; however, accumulating reports of familial cases of P-NSAH and nP-NSAH raise the question of whether there are genetic factors that play an important role in the development of SAH [6,7].

We report the cases of two siblings with P-NSAH, which suggest first-degree relatives of individuals with this entity may have a higher risk for SAH.

## Case presentation

### Case 1

**Figure 1:**
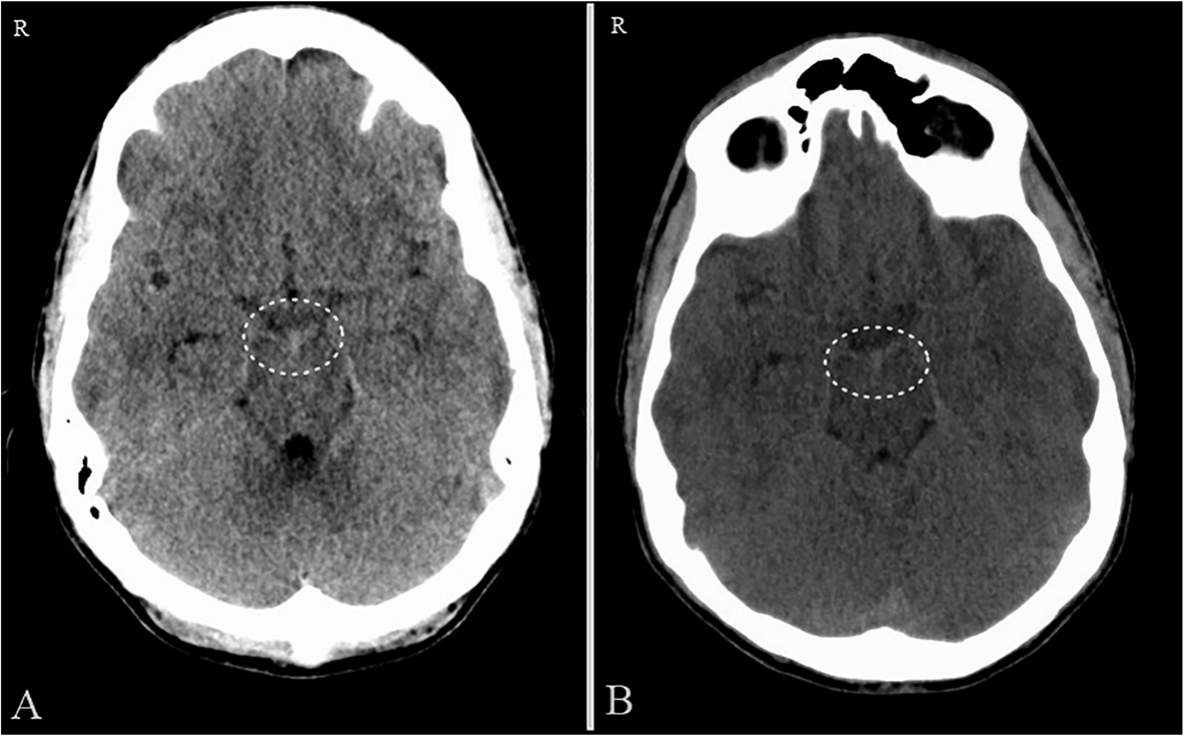
**Case 1.** Axial non-contrast computed tomography of the head **(A and B)** shows a small amount of subarachnoid blood (circle) in the interpeduncular and ambient cisterns.

**Figure 2:**
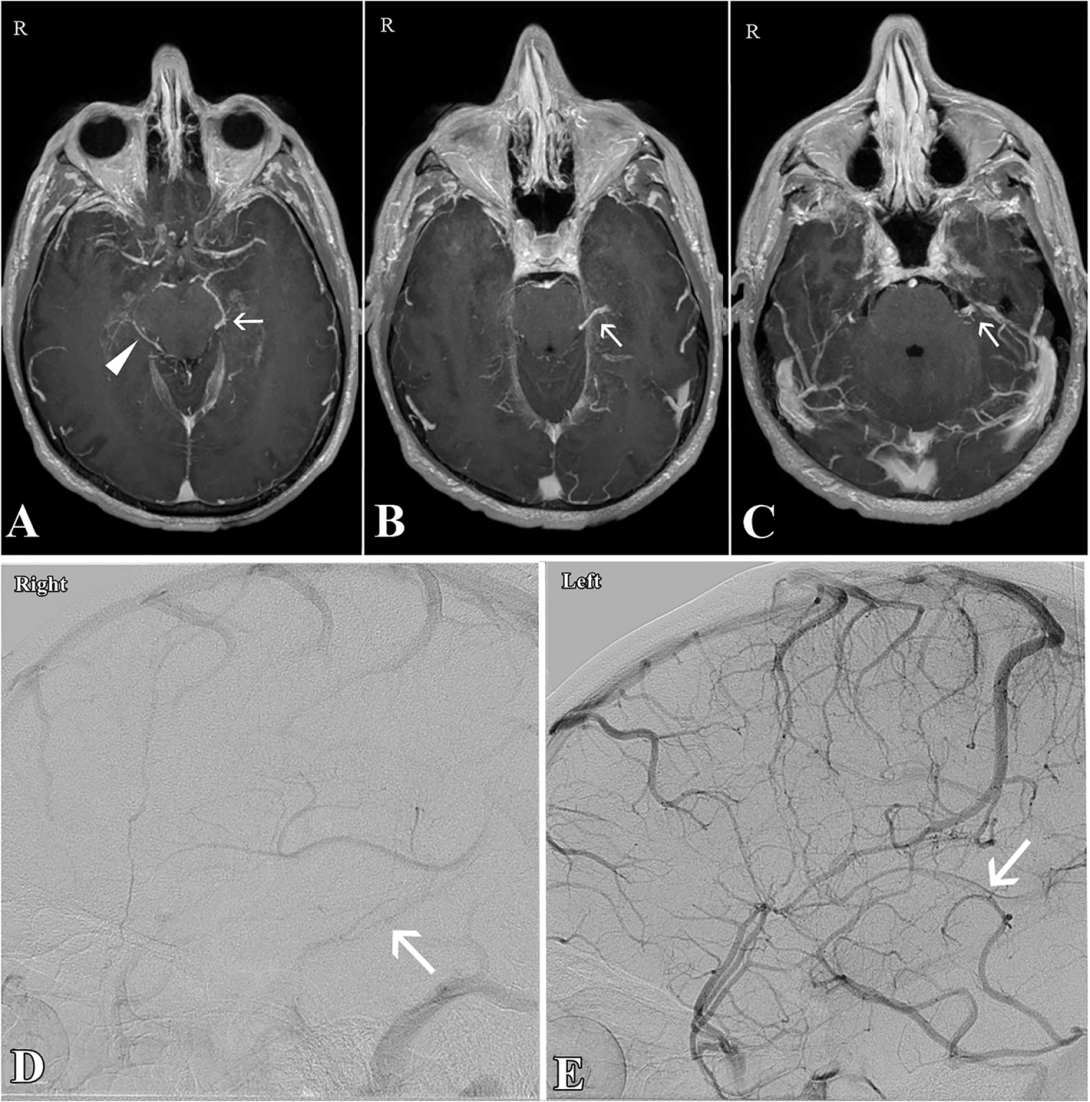
**Case 1.** Axial magnetic resonance angiography **(A, B and C)** shows a Type A basal vein of Rosenthal on the right side (arrow head in **A**) and a Type C basal vein of Rosenthal on the left side (arrows in **A**, **B** and **C**). Lateral view of the venous phase of the internal carotid artery angiogram **(D and E)** shows a hypoplastic Type A basal vein of Rosenthal on the right side (arrow in **D**) and a Type C basal vein of Rosenthal draining into the superior petrosal sinus on the left side (arrow in **E**).

A 39-year-old otherwise healthy Caucasian man presented with spontaneous, sudden onset headache associated with diplopia lasting for several minutes. The most severe headache episode lasted for about 15 minutes. During admission, the intensity of his headaches diminished significantly along with an improvement of the diplopia. On admission, he was awake, alert and fully oriented with fluent speech. He had no focal neurological deficits except nuchal rigidity. He was a non-cigarette smoker with no significant past medical history. A CT of his head without contrast revealed subtle subarachnoid hemorrhage within the interpeduncular and ambient cisterns (Figure 1A and 1B). A subsequent lumbar puncture was found positive.He was admitted to our neurosurgical intensive care unit (N-ICU) for observation and further work-up. Coagulation studies were normal. Further evaluation with magnetic resonance imaging and angiography of his head and neck and 4-vessel cerebral angiogram did not show any abnormalities (Figure 2A-2C). Venous phases of the cerebral angiograms showed a Type C variant of the basal vein of Rosenthal (BVR) draining into the superior petrosal sinus on the left side and a small and hypoplastic Type A variant of BVR on the right side (Figure 2D and 2E). His symptoms improved and eventually he was discharged 1 week after the bleeding. On follow-up at 2 years, no neurological deficit and no recurrent subarachnoid hemorrhage were observed.

### Case 2

**Figure 3:**
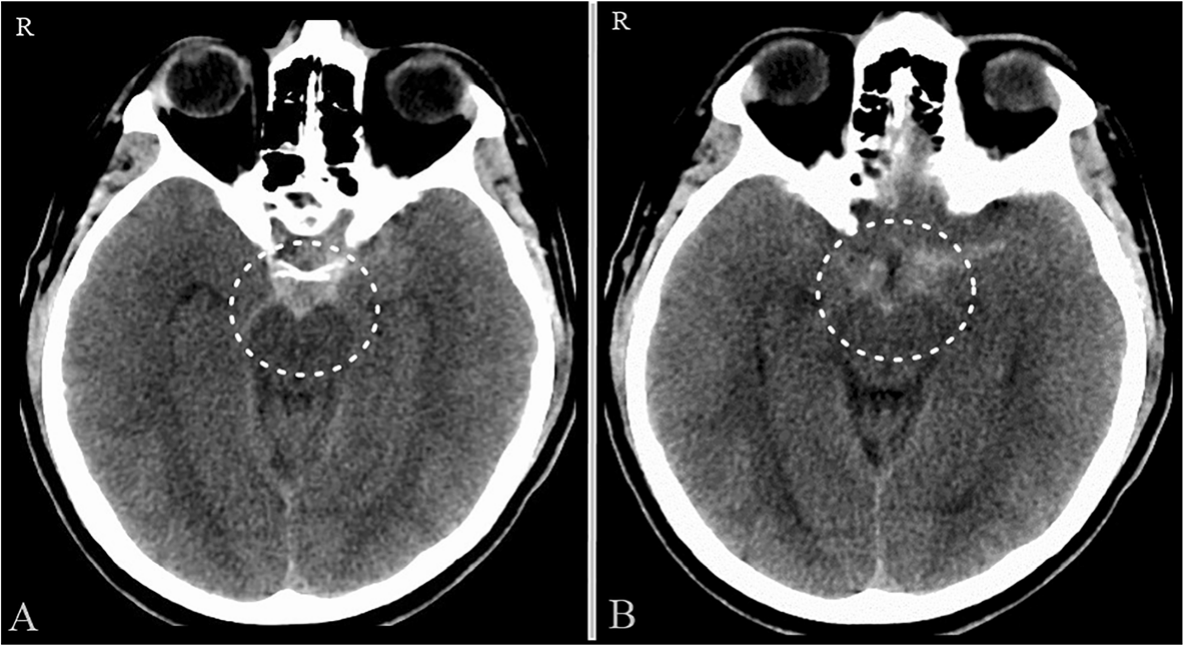
**Case 2.** Axial non-contrast computed tomography of the head **(A and B)** shows perimesencephalic spontaneous subarachnoid hemorrhage (circle) with the center of bleeding located anterior to the midbrain.

**Figure 4:**
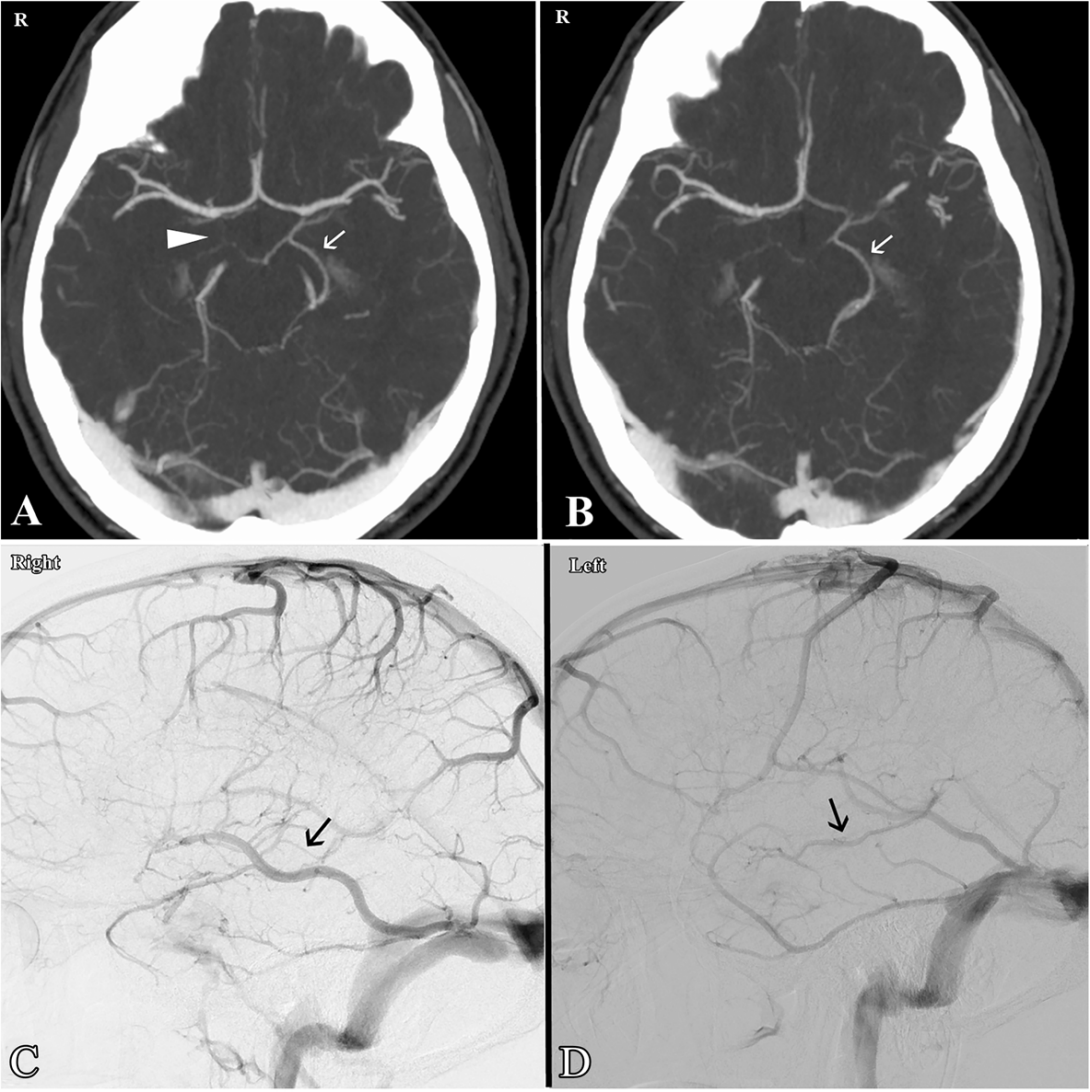
**Case 2.** Axial computed tomography angiography of the head **(A and B)** shows a Type B basal vein of Rosenthal on the right side (arrow head in **A**) and a Type A basal vein of Rosenthal on the left side (arrows in **A** and **B**). Lateral view of the venous phase of the internal carotid artery angiogram **(C and D)** shows a Type B basal vein of Rosenthal on the right side (arrow in **C**; the basal vein of Rosenthal is not visualized anteriorly) and a Type A basal vein of Rosenthal on the left side (arrow in **D**).

A 44-year-old otherwise healthy Caucasian man, the older brother of the first case, presented with the spontaneous acute onset of headache. He denied loss of consciousness, vision loss, nausea, vomiting, numbness, weakness and dizziness. On neurological examination, he was awake, alert and fully oriented with fluent speech. In the presence of slight nuchal rigidity, he had full motor strength with no sensory deficit in all four extremities and his cranial nerves were intact. He was a non-cigarette smoker and had no significant medical history except inguinal hernia repair. Family history was positive for P-NSAH in younger brother (Case 1), which was diagnosed 1.5 years previously. A non-contrast axial CT scan of his head revealed a perimesencephalic SAH pattern and no evidence of intraventricular or intraparenchymal blood and hydrocephalus. The center of bleeding was located anterior to the midbrain (Figure 3A and 3B).He was admitted to our N-ICU for observation and further work-up. No abnormality was found in the coagulation studies. He underwent additional multiple imaging studies, including CT and CT angiography of his head and neck (Figure 4A and 4B) and 4-vessel cerebral angiograms. None of these diagnostic studies showed the source of this SAH. Venous phases of the angiograms showed a Type A variant of BVR on the left side and a Type B variant of BVR on the right side (Figure 4C and 4D). His symptoms continued to improve and eventually he was discharged 1 week after the bleeding. A repeat cerebral angiogram 3 months later again confirmed the absence of any aneurysm and vascular abnormality. On 1 year follow-up, his neurological examination was normal and no recurrent subarachnoid hemorrhage was observed.

## Discussion

P-NSAH was first described by van Gijn *et al.*[2] and characterized by subarachnoid blood predominantly around the mesencephalon without any vascular pathology or any other source of bleeding [2,3]. The typical CT pattern of P-NSAH has been described by Rinkel *et al*. as follows: 1) subarachnoid blood around the mesencephalon, with possible extension to the posterior part of the interhemispheric fissure without complete filling of the fissure; 2) extension of the subarachnoid blood to the basal part of the sylvian cistern; 3) minimal sedimentation of intraventricular blood; 4) absence of intraventricular blood [8]. While both arterial and venous origins have been postulated as the source of hemorrhage, the precise etiology of P-NSAH remains enigmatic despite extensive diagnostic investigation [1,3]. A non-arterial origin has been proposed in the literature due to infrequent loss of consciousness, lack of blood in the brain parenchyma or ventricular system, and low incidence of vasospasm and hydrocephalus. Furthermore, accumulation of subarachnoid blood around the brainstem has drawn the attention of investigators to variations of the perimesencephalic and deep cerebral veins [8]. Typically, blood is found in the interpeduncular, crural and ambient cisterns, all of which have a close relationship with the BVR.

A growing body of evidence has supported the role of anatomic variations of this vein as a critical contributor to P-NSAH pathogenesis [9-12]. Watanabe *et al.* studied the relationship between P-NSAH and variations of deep cerebral veins in particular BVR and classified the venous drainage of BVR into three types [12]. Type A is the normal continuous pattern, in which the BVR is continuous with the deep middle cerebral vein and drains mainly to the vein of Galen (VG). Type B is a normal discontinuous pattern with drainage anterior to uncal veins and posterior to VG. Type C is a primitive variant with drainage to other veins but not to the VG. In a study by Watanabe *et al.*, Type C variants of the BVR on one or both sides were identified in all patients with P-NSAH . Moreover, the Type C pattern was found much more frequently in patients with P-NSAH than in patients with aneurysmal SAH [12]. Similar findings have been later reported by different groups [9-11]. In these studies, in addition to Type C, Type B also was found to be associated with P-NSAH more frequently than SAH [9,10]. All venograms of our two patients have been retrospectively reviewed by two separate neuroradiologists who confirmed the presence of a Type C variant on the left side in patient 1 and a Type B variant on the right side in patient 2. Both patients had Type A on the contralateral side (Figures 2D, 2E and 4C, 4D).

A series of reports have highlighted the critical role of genetic factors in the pathogenesis of aneurysmal SAH [4-7,13]. A large population-based study by Bor *et al.* revealed that people with two first-degree relatives affected with SAH have a higher risk of SAH in comparison with individuals with just one affected first-degree relative [4]. Furthermore, Bromberg *et al.* have shown that the SAH occurrence in first-degree relatives is up to seven times greater than in second-degree relatives [13]. It is suggested that the first-degree relatives of patients with aneurysmal SAH are at a higher risk of bleeding than the general population, similar results for NSAH have not been reported however [4,5,13].

Although the etiology of NSAH has not been determined, some risk factors for NSAH have been defined, such as: female sex, arterial hypertension, smoking, and excessive use of alcohol [14,15]. There are only two case reports describing an association between the familial predisposition of venous system variants and NSAH in the literature [6,7]. Type C variant of the BVR has been reported in two first-degree relatives with P-NSAH [7] (Table 1); however, three siblings with NSAH (one with nP-NSAH, two of them with P-NSAH) have been described with no report of BVR variant [6].

**Table 1:** Spontaneous perimesencephalic subarachnoid hemorrhage in siblings

**Authors**		**Age**	**Gender**	**Complaint at presentation**	**Risk factors for spontaneous subarachnoid hemorrhage**	**Follow up**	**Basal vein of Rosenthal (right/left)**
**Tielman** ***et al****.*[7]	Patient 1	51 years	Man	Headache, vomiting, left gaze diplopia	No	No recurrent subarachnoid hemorrhage and no neurological deficits	Type C/Type C
	Patient 2	50 years	Woman	Headache, loss of consciousness, vomiting	No	No recurrent subarachnoid hemorrhage and no neurological deficits	Type C/Type C
**Cikla** ***et al****.***(this article)**	Patient 1	39 years	Man	Headache, diplopia, nuchal rigidity	No	No recurrent subarachnoid hemorrhage and no neurological deficits	Type A/Type C
	Patient 2	44 years	man	Headache, slight nuchal rigidity	No	no recurrent subarachnoid hemorrhage and no neurological deficits	Type B/Type A

Our report is the second familial occurrence of P-NSAH in two siblings with Type B and Type C BVR variants.

## Conclusions

In a concise manner, our report together with the previous reports [6,7] suggest that a positive family history in first-degree relatives of P-NSAH accounts for higher risk of P-NSAH. Further investigation, however, is necessary to illuminate the precise role of familial inheritance in pathogenesis. In addition, reporting new aspects of such rare associations would help physicians to better understand the underlying pathology in these types of inadequately explained clinical entities.

## Consent

Written informed consent was obtained from the patients for publication of this case report and any accompanying images. Copies of the written consents are available for review by the Editor-in-Chief of this journal.

## Abbreviations

BVR: Basal vein of Rosenthal; CT: Computed tomography; N-ICU: Neurosurgical intensive care unit; nP-NSAH: Non-perimesencephalic non-aneurysmal subarachnoid hemorrhage; NSAH: Non-aneurysmal subarachnoid hemorrhage; P-NSAH: Perimesencephalic non-aneurysmal subarachnoid hemorrhage; SAH: Spontaneous subarachnoid hemorrhage; VG: Vein of Galen.

## Competing interests

The authors declare that they have no competing interests.

## Authors’ contributions

UC, MKB, DBN and GM designed the study, drafted and revised the manuscript. BAK and PAT were responsible for radiological assessment. All authors read and approved the final manuscript.

## References

[B1] Canovas D Gil A Jato M Miqueld M Rubio F Clinical outcome of spontaneous non-aneurysmal subarachnoid hemorrhage in 108 patients Eur J Neurol 2012 19 457 461 10.1111/j.1468-1331.2011.03542.x 21972883

[B2] Van Gijn J van Dongen KJ Vermeulen M Hijdra A Perimesencephalic hemorrhage: a nonaneurysmal and benign form of subarachnoid hemorrhage Neurology 1985 35 493 497 10.1212/WNL.35.4.493 3982634

[B3] Boswell S Thorell W Gogela S Lyden E Surdell D Angiogram-negative subarachnoid hemorrhage: outcomes data and review of the literature J Stroke Cerebrovasc Dis 2013 22 750 757 10.1016/j.jstrokecerebrovasdis.2012.02.001 22465208

[B4] Bor AS Rinkel GJ Adami J Koffijberg H Ekbom A Buskens E Blomqvist P Granath F Risk of subarachnoid haemorrhage according to number of affected relatives: a population based case-control study Brain 2008 131 2662 2665 10.1093/brain/awn187 18819992

[B5] Korja M Silventoinen K McCarron P Zdravkovic S Skytthe A Haapanen A de Faire U Pedersen NL Christensen K Koskenvuo M Kaprio J GenonEUtwin Project Genetic epidemiology of spontaneous subarachnoid hemorrhage: Nordic Twin Study Stroke 2010 41 2458 2462 10.1161/STROKEAHA.110.586420 20847318

[B6] Lazaridis C Bodle J Chaudry I Hays A Chalela J Familial nontraumatic, nonaneurysmal subarachnoid hemorrhage: a report on three first-degree siblings J Neurosurg 2011 115 621 623 10.3171/2011.5.JNS1119 21639697

[B7] Tieleman AA van der Vliet TA Vos PE Two first-degree relatives with perimesencephalic nonaneurysmal hemorrhage Neurology 2006 67 535 536 10.1212/01.wnl.0000227921.50128.55 16894127

[B8] Rinkel GJ Wijdicks EF Hasan D Kienstra GE Franke CL Hageman LM Vermeulen M van Gijn J Outcome in patients with subarachnoid haemorrhage and negative angiography according to pattern of haemorrhage on computed tomography Lancet 1991 338 964 968 10.1016/0140-6736(91)91836-J 1681340

[B9] Alen JF Lagares A Campollo J Ballenilla F Kaen A Nunez AP Lobato RD Idiopathic subarachnoid hemorrhage and venous drainage: are they related? Neurosurgery 2008 63 1106 1111 10.1227/01.NEU.0000335777.14055.71 19057322

[B10] Sabatino G Della Pepa GM Scerrati A Maira G Rollo M Albanese A Marchese E Anatomical variants of the basal vein of Rosenthal: prevalence in idiopathic subarachnoid hemorrhage Acta Neurochir 2014 156 45 51 10.1007/s00701-013-1907-6 24136678

[B11] van der Schaaf IC Velthuis BK Gouw A Rinkel GJ Venous drainage in perimesencephalic hemorrhage Stroke 2004 35 1614 1618 10.1161/01.STR.0000131657.08655.ce 15166390

[B12] Watanabe A Hirano K Kamada M Imamura K Ishii N Sekihara Y Suzuki Y Ishii R Perimesencephalic nonaneurysmal subarachnoid haemorrhage and variations in the veins Neuroradiology 2002 44 319 325 10.1007/s00234-001-0741-3 11914808

[B13] Bromberg JE Rinkel GJ Algra A Greebe P van Duyn CM Hasan D Limburg M ter Berg HW Wijdicks EF van Gijn J Subarachnoid haemorrhage in first and second degree relatives of patients with subarachnoid haemorrhage BMJ 1995 311 288 289 10.1136/bmj.311.7000.288 7633233 PMC2550356

[B14] Canhao P Falcao F Pinho e Melo T Ferro H Ferro J Vascular risk factors for perimesencephalic nonaneursymal subarachnoid hemorrhage J Neurol 1999 246 492 496 10.1007/s004150050389 10431777

[B15] Connoly ES Jr Choudhri TF Mack WJ Mocco J Spinks TJ Slosberg J Lin T Huang J Solomon RA Influence of smoking, hypertension, and sex on the phenotypic expression of familial intracranial aneurysms in siblings Neurosurgery 2001 48 64 68 discussion:68–69 11152362 10.1097/00006123-200101000-00011

